# Why do people comply with health guidelines in a competing information environment?

**DOI:** 10.1371/journal.pone.0352421

**Published:** 2026-06-26

**Authors:** Jiadong Yu, D.A. Bekerian, Radhika Khandelwal

**Affiliations:** 1 Department of Psychology, Georgia Southern University, Statesboro, Georgia, United States of America; 2 California School of Professional Psychology, Alliant University, Fresno, California, United States of America; Southwest Petroleum University, CHINA

## Abstract

The COVID-19 pandemic unfolded in parallel with an infodemic where competing information from formal and informal sources shaped protective behavior. This study conducted a secondary analysis of cross-sectional survey data from 742 U.S. adults to examine how reliance on different information channels influenced risk perceptions, socio-political beliefs, and protective intentions. Structural equation modeling revealed that reliance on formal sources, such as government websites and mainstream news, was positively associated with perceived severity, reduced conspiracy endorsement, and more liberal political orientation, all of which increased mask-wearing and vaccination intentions. Reliance on informal sources, including social media and peer networks, reduced severity perceptions, heightened conspiracy beliefs, and reinforced more conservative orientations, which in turn undermined compliance. Perceived severity functioned as a central mediator, directly encouraging mask use and indirectly promoting vaccination through reduced conspiracy endorsement. Political ideology and conspiracy beliefs further shaped vaccine intentions, highlighting the ideological pathways through which information exerts influence. Mask-wearing and vaccination clustered together, indicating a generalized orientation toward protective action. The model underscored the critical role of information environments as upstream determinants of health behavior during crises. These findings emphasize that managing both formal and informal channels is essential for effective crisis communication and for strengthening public compliance in future health emergencies.

## Introduction

The COVID-19 pandemic was not only one of the most disruptive health crises of the 21st century but also a defining infodemic. As Covid-19 spread globally, the circulation of information about prevention, treatment, and vaccines was as consequential as the virus itself. While public health authorities issued evidence-based recommendations, competing narratives proliferated on social media, peer networks, and informal platforms. This dual pandemic of disease and misinformation created conditions in which health decisions became less about biomedical facts and more about which sources of information individuals trusted [1–3].

Early in the pandemic, policymakers and scholars drew upon decades of health psychology research to interpret protective behavior. The Health Belief Model (HBM; [4]), Protection Motivation Theory (PMT; [5]), and the Theory of Planned Behavior (TPB; [6]) provided dominant theoretical lenses for anticipating compliance. Each of these models highlights cognitive determinants of health action: perceived severity, perceived susceptibility, perceived benefits, barriers, self-efficacy, and normative pressures. Within threat-appraisal frameworks in particular, perceived risk is often conceptualized not as two entirely separate indicators, but as a broader perceived threat construct that combines judgments of susceptibility and severity. Consistent with this view, prior work has treated perceived threat as the joint appraisal of how likely a health threat is and how serious its consequences may be, sometimes operationalizing it through a composite or interaction of perceived susceptibility and perceived severity [7]. Empirical research confirmed that individuals who viewed COVID-19 as highly severe and who perceived themselves as vulnerable were more likely to adopt protective behaviors such as hand hygiene, mask-wearing, and vaccination [8–10]. These findings reinforced the value of traditional cognitive models, showing that risk appraisals continue to play a crucial role in shaping health decisions during crises [11].

However, as the pandemic progressed, it became increasingly clear that cognitive appraisals alone could not fully account for behavioral variability. Two individuals with similar perceptions of severity and vulnerability could arrive at very different choices depending on their political orientation, trust in institutions, and the sources from which they consumed information. In the United States, partisanship strongly predicted mask-wearing and vaccination intentions even after controlling for perceived risk [12]. In politically polarized environments, people actively seek positive information regarding their political party and are less capable of perceiving and remembering negative information regarding their party, making it so that, over time, they begin to identify with that party and develop in-group perceptions. Group identity, rather than objective risk, becomes the primary lens through which individuals interpret health information. Rather than basing threat appraisals on epidemiological data, individuals may interpret health information through cues that align with their ideological identity [13,14]. Conservatives were significantly less likely to comply with public health guidelines than liberals, often framing protective measures as threats to individual freedom rather than collective responsibility [15,16]. Ideological differences were often linked to divergent trust in scientific expertise and public health institutions, which in turn influence perceived threat and beliefs about the effectiveness of recommended actions [17]. Simultaneously, conspiracy beliefs proliferated, ranging from claims that the virus was engineered for political gain to suspicions that vaccines were unsafe or intentionally harmful [18–20]. These beliefs eroded trust in scientific authorities and undermined adherence to guidelines, illustrating the importance of ideological and cultural factors in shaping responses to health crises [21].

Central to these processes was the information environment. Unlike earlier pandemics, COVID-19 unfolded in a digital age where social media, blogs, podcasts, and peer-to-peer networks amplified alternative interpretations of events. Formal channels, government health agencies, mainstream news outlets, and official reports, competed directly with informal channels that were often more accessible, more personalized, and, in some cases, more appealing to individuals predisposed toward skepticism of authority [22–24]. Research consistently shows that reliance on formal sources is positively associated with compliance, whereas reliance on informal sources is associated with lower adherence, greater susceptibility to conspiracy theories, and political polarization [2,25,26]. For example, in Austria, individuals who reported social media as their main COVID-19 information source showed consistently lower agreement with non-pharmaceutical interventions during the first lockdown [25]. Similarly, longitudinal evidence from China demonstrated that trust in government and reliance on official channels predicted greater sustained adherence to protective behaviors [27].

Health communication theories underscore the mechanisms by which information exposure influences behavior. The Risk Information Seeking and Processing (RISP) model [28] highlights how individuals’ perceptions of risk and efficacy shape their motivation to seek and process information, which then informs decision-making. The Extended Parallel Process Model (EPPM; [29]) emphasizes the interaction of threat and efficacy in determining whether fear appeals result in adaptive or maladaptive outcomes. During COVID-19, for example, fear-based messages sometimes prompted compliance but, in polarized contexts, also reinforced avoidance or motivated individuals to embrace conspiracy explanations as coping strategies [30,31]. Together, these models demonstrate that information channels are not passive conduits; they actively filter, frame, and reshape risk appraisals and behavioral responses.

However, the distinction between formal and informal sources is not simply a matter of content accuracy but also of trust, identity, and interpretive frames. Formal sources are embedded within institutional credibility; they rely on expertise, evidence, and consistency [32]. Informal sources are situated within community ties and digital ecosystems; they carry the weight of interpersonal trust, social reinforcement, and identity signaling [33]. During COVID-19, these two informational pathways generated divergent realities. For many, exposure to formal channels heightened perceived severity and reinforced the collective importance of protective action [34]. For others, immersion in informal channels normalized skepticism, trivialized risks, and amplified alternative explanations [35]. Such dynamics illustrate how information channels operate as upstream determinants of health behavior, shaping the cognitive and socio-political variables that models like HBM, PMT, and TPB have historically emphasized.

Previous work, including our own integrative model [36], examined how cognitive appraisals, political ideology, and conspiracy beliefs jointly predicted protective intentions. That study underscored the importance of socio-political variables, showing that ideology and conspiracies can override traditional cognitive predictors of health decisions. Yet, the role of information sources was addressed only as one of several factors rather than the focal driver. The current study builds directly on that foundation by reframing information sources as the starting point of a causal cascade. By distinguishing between reliance on formal versus informal channels, we examine how these sources influence risk perceptions, foster or suppress conspiracy beliefs, shape political orientation, and ultimately determine intentions to wear masks and receive vaccinations. This reframing is important because protective behaviors are rarely isolated. Evidence shows that individuals who engage in one preventive behavior, such as mask-wearing, are more likely to engage in others, such as vaccination, suggesting a clustering of compliance [37,38].

By situating information sources at the top of the decision chain, the present study provides an explanatory framework for why compliance behaviors cluster and why resistance can generalize across multiple guidelines. The specific objectives of this study are fourfold. First, we seek to examine the relationship between reliance on formal versus informal sources of health information and individuals’ perceptions of severity and vulnerability to COVID-19. Second, we investigate how reliance on these information sources influences socio-political variables, specifically political ideology and conspiracy beliefs. Third, we test whether perceived severity, perceived vulnerability, political ideology, and conspiracy beliefs mediate the relationship between information sources and protective behaviors. Finally, we evaluate the interrelationship between protective intentions themselves, specifically whether willingness to wear masks predicts willingness to vaccinate. Collectively, these objectives enable a comprehensive examination of the mechanisms through which information environments shape health behaviors during a public health crisis.

## Materials and methods

### Study design

The present study is a secondary analysis of cross-sectional survey data collected in the United States between 28 and 30 January 2023, as part of a larger project on compliance with COVID-19 health guidelines [36]. While the earlier publication examined cognitive appraisals, conspiracy beliefs, and political ideology, the current analysis focuses specifically on the role of information sources as upstream determinants of protective intentions. By reframing the analytic model, this study addresses a distinct set of objectives concerning how reliance on formal versus informal channels shapes perceived severity, perceived vulnerability, conspiracy beliefs, political ideology, and subsequent behavioral intentions.

### Participants

Participants were recruited through Amazon Mechanical Turk (MTurk), an online crowdsourcing platform, using a nonprobability convenience sampling approach. Eligibility criteria required individuals to be at least 18 years old and to have sufficient English proficiency to complete the survey. Informed consent was obtained electronically prior to participation, and compensation was provided at a fixed rate. One attention-check item was embedded in the survey to enhance data quality; participants who failed this item were excluded from further analyses. The target sample size was determined through a priori power analysis with α = .05 and power = .80, to ensure adequate power for the planned structural equation modeling [39].

The original data collection protocol received approval from the Institutional Review Board of Alliant International University (Protocol Number: 2203238687A002), and all procedures were performed in accordance with relevant ethical standards and reporting guidelines. The current study was conducted as a secondary analysis of data originally collected under that protocol.

### Measures

All study constructs were measured with multi-item Likert-type scales using a seven-point response format ranging from 1 (strongly disagree) to 7 (strongly agree). Higher scores consistently reflected stronger endorsement of the construct in question. Measurement items were adapted from the original study by Yu and Bekerian [36]. In the present study, formal and informal information sources were operationalized as broad source categories based on the typical origin and structure of the information rather than as fully discrete or mutually exclusive information environments. Formal sources referred primarily to institutionally produced or officially sanctioned channels, whereas informal sources referred to socially distributed or less institutionally regulated channels. Reliance on formal information sources was measured with six items addressing the use of official channels such as government health websites (Cronbach’s α = .86) [36]. Reliance on informal sources was measured with five items referencing social media and information from family, friends, and colleagues (Cronbach’s α = .91) [36].

Perceived severity was measured with five items assessing the extent to which participants regarded COVID-19 infection as serious and threatening (Cronbach’s α = .91) [36]. Perceived vulnerability was measured with five items capturing respondents’ sense of personal susceptibility relative to others of similar age (Cronbach’s α = .89) [36]. Conspiracy beliefs were assessed with seven items capturing endorsement of common narratives that portrayed the virus as intentionally engineered or manipulated for political or economic purposes (Cronbach’s α = .96) [36].

Political orientation was assessed using five items reflecting participants’ ideological self-identification and partisan alignment, where higher scores reflected stronger liberal alignment (Cronbach’s α = .97) [36]. Because some items referred to liberal-conservative orientation, whereas others referred to Democratic versus Republican preference, this construct should be interpreted as capturing a broader sociopolitical orientation rather than a pure measure of ideology alone. Protective intentions were measured in two domains: mask-wearing intentions, assessed with four items concerning the likelihood of mask use in different public contexts (Cronbach’s α = .96), and vaccination intentions, assessed with four items addressing willingness to obtain initial or booster vaccinations under various recommendation conditions (Cronbach’s α = .95) [36]. All multi-item scales demonstrated strong internal consistency in this sample, with Cronbach’s alpha coefficients exceeding 0.80.

### Data Analysis

**Fig 1:**
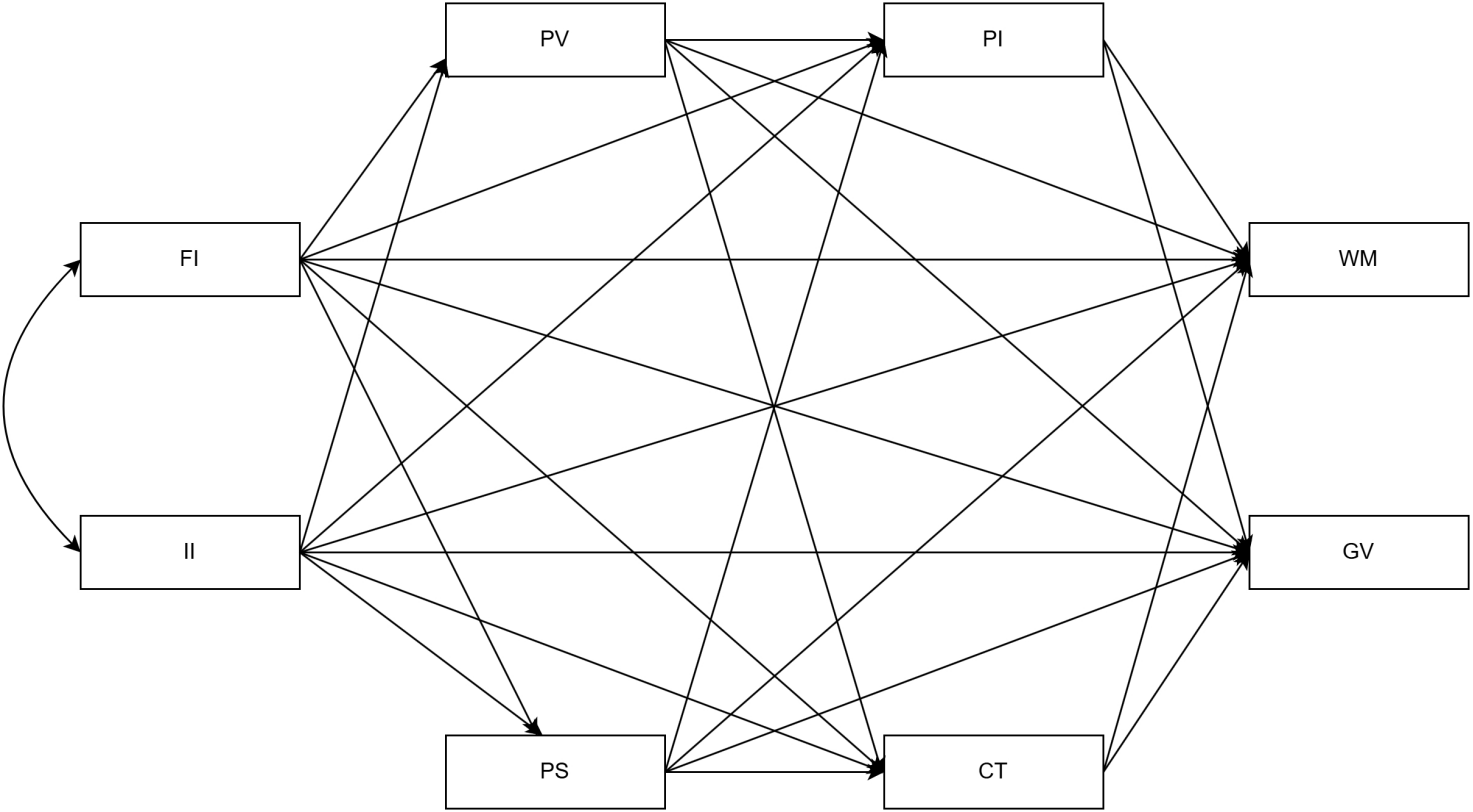
A Conceptual Model for Understanding Sources of Health Guidelines, Beliefs, and Compliances. *Note.* FI: Formal information, II: Informal information, PV: Perceived vulnerability, PS: Perceived severity, PT: Political ideology, CT: Conspiracy theories, WM: Willingness to wear masks, GV: Intention to get vaccinated.

Analyses were conducted in SPSS version 27.0 and AMOS version 29.0. Preliminary checks confirmed that there were no problematic outliers or departures from normality. The primary analytic strategy was structural equation modeling (SEM) to evaluate the hypothesized relationships among information sources, health belief constructs, and protective behavioral intentions (Fig 1). Models were estimated using maximum likelihood estimation [40]. Path coefficients with p-values less than 0.05 were deemed statistically significant. Model adequacy was assessed using several indices, including the Comparative Fit Index (CFI), the Tucker–Lewis Index (TLI), and the Root Mean Square Error of Approximation (RMSEA). CFI and TLI values above 0.90 and RMSEA values below 0.08 were considered indicative of acceptable fit [40–43].

## Results

**Fig 2:**
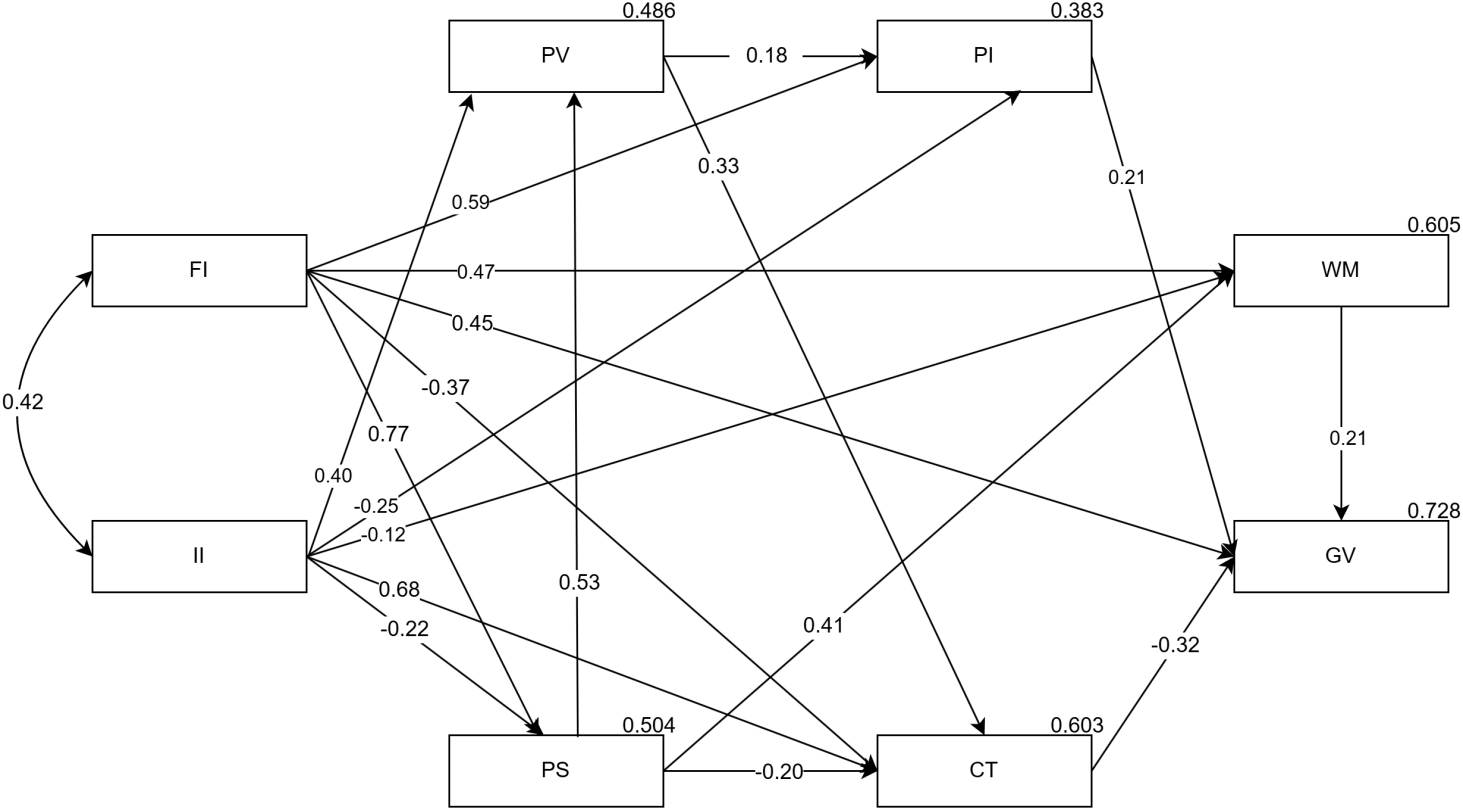
A Modified Model for Understanding Sources of Health Guidelines, Beliefs, and Compliances. *Note.* FI: Formal information, II: Informal information, PV: Perceived vulnerability, PS: Perceived severity, PT: Political ideology, CT: Conspiracy theories, WM: Willingness to wear masks, GV: Intention to get vaccinated.

Of the 755 individuals who initiated the survey, a total of 742 participants provided complete data and were retained for analysis. Respondents were on average 39.5 years old (SD = 12.3), and 53.5% identified as female. The dataset used and analyzed during the current study is available within the supporting information files. The hypothesized Model I (Fig 1) was initially tested with all paths from sources of information to risk perceptions and beliefs, and further on to protective behaviors freely estimated. However, it yielded inadequate fit indices, χ² (7) = 391.74, CFI = 0.877, TLI = 0.510, RMSEA = 0.272. Subsequently, nonsignificant paths (p ≥ 0.05) were eliminated from Model I, resulting in a refined version (Model II). Model II (Fig 2) was then reanalyzed to assess its overall fit to the data. A comparison of model fit indices (Table 1) indicated that Model II achieved a notably better fit, χ²(9) = 36.7, CFI = 0.993, TLI = 0.978, RMSEA = 0.064, indicating that the trimmed specification provided a more accurate representation of the data. The revised model showed improved fit after the removal of nonsignificant paths; however, this refined model should not be interpreted as a theoretically final representation of the proposed relationships. Model modification based on the present sample reflected sample-specific characteristics.

**Table 1:** Comparison of model fit indices of Model I and Model II.

	χ2 (df)	CFI	TLI	RMSEA	p-value
Model I	391.744 (7)	0.877	0.510	0.272	<.001
Model II	51.593 (11)	0.987	0.967	0.071	<.001

Reliance on formal information sources was strongly and positively associated with perceived severity (β = .775, p < .001), whereas reliance on informal information sources showed a negative association with severity (β = −.224, p < .001). Perceived vulnerability increased with greater severity (β = .531, p < .001) and with reliance on informal information (β = .400, p < .001). Conspiracy beliefs were lower when severity was higher (β = −.200, p < .001) and when people relied on formal information (β = −.371, p < .001) but were higher with greater reliance on informal information (β = .679, p < .001) and with greater vulnerability (β = .326, p < .001). Political ideology (higher = more liberal) increased with formal information (β = .587, p < .001) and with vulnerability (β = .177, p < .001) and decreased with informal information (β = −.254, p < .001).

Mask-wearing intention was higher among those perceiving greater severity (β = .406, p < .001), higher among those relying on formal information (β = .471, p < .001), and lower among those relying on informal information (β = −.117, p < .001). Vaccination intention increased with more liberal political ideology (β = .206, p < .001), with reliance on formal information (β = .454, p < .001), and with stronger mask-wearing intention (β = .210, p < .001), and decreased with stronger conspiracy beliefs (β = −.317, p < .001).

Overall, Model II explained 48.6% of the variance in perceived vulnerability, 50.4% of the variance in perceived severity, 38.3% of the variance in political ideology, 60.3% of the variance in conspiracy theory beliefs, 60.5% of the variance in intentions to wear masks, and 72.8% of the variance in intentions to get vaccinated.

## Discussion

This study applied SEM to clarify how formal and informal sources of health information shaped risk perceptions, socio-political beliefs, and intentions to engage in protective behaviors during the COVID-19 pandemic. By reframing information sources as upstream determinants rather than peripheral variables, the analysis highlights the pathways through which reliance on different information channels cascades into distinct cognitive and ideological patterns, which in turn predict intentions to wear masks and vaccinate. All four sets of objectives were examined, providing consistent evidence that formal and informal sources of information generate opposing belief trajectories with consequential effects for health guideline adherence.

The first objective was to examine the relationship between information sources and perceptions of severity and vulnerability. Results indicated that reliance on formal sources such as government websites, mainstream television, and official reports was strongly associated with higher perceived severity, while reliance on informal sources such as social media, blogs, and peer networks was associated with lower severity and heightened vulnerability. These findings extend the core predictions of models such as the HBM and PMT, which posit that risk appraisals are central to protective action [4,5]. The results demonstrate that risk appraisals themselves are products of information ecosystems. This helps explain why, during COVID-19, populations with similar epidemiological exposures arrived at divergent perceptions of risk: they were consuming information from different channels, with formal sources emphasizing seriousness and informal sources often trivializing threats.

The second objective was to investigate whether information sources shaped conspiracy beliefs and political ideology. Reliance on formal sources was linked to lower conspiracy endorsement and to more liberal political alignment, whereas reliance on informal sources was linked to stronger conspiracy endorsement and more conservative orientations that were less supportive of public health mandates. These findings are consistent with previous evidence showing that conspiracy beliefs undermine compliance [18,19] and that political partisanship conditions acceptance of guidelines [15]. What the present study adds is evidence that political ideology and conspiracy beliefs are not merely individual differences but are systematically patterned by reliance on different types of information sources. Informal channels provide fertile ground for conspiracy narratives and identity-based resistance, while formal channels was associated with greater alignment with institutionally endorsed public health responses.

The third objective was to test indirect pathways, probing how information sources exert influence on behavioral intentions indirectly through severity perceptions and political ideology. Both indirect pathways were significant, though in each case, mediation was partial, suggesting that formal sources affect intentions both directly and indirectly. For mask-wearing intentions, severity appraisals carried a substantial portion of the effect: individuals who relied on formal sources viewed COVID-19 as more serious, which increased their willingness to wear masks. Yet the direct effect of formal source use on mask-wearing remained robust, indicating that messaging from credible institutions also encourages compliance independent of cognitive appraisal. For vaccination, political ideology served as a mediator: reliance on formal sources predicted more liberal orientations, which in turn predicted greater willingness to vaccinate. Again, both direct and indirect effects were significant. These findings refine existing theories by showing that protective behaviors are not driven only by perceived health threats, but also by socio-political alignments that are themselves shaped by information ecosystems. The results confirm the theoretical integration of health behavior models with health communication perspectives such as the RISP framework [28] and the EPPM [29]. Information channels do not simply deliver content; they influence both the appraisals and the ideological frames through which content is interpreted, thereby altering compliance behavior.

The fourth objective is to test whether protective behaviors would cluster, such that intentions to wear masks would be positively associated with intentions to vaccinate. This prediction was confirmed, supporting the idea that compliance reflects a general orientation toward protective action rather than isolated, behavior-specific choices. This finding aligns with prior studies reporting correlations between adherence to different non-pharmaceutical interventions [37,38]. The present results extend this evidence by embedding clustering within a larger structural model: reliance on formal information sources not only increases compliance with specific behaviors but also strengthens a generalized compliance orientation.

One unexpected finding was the positive association between perceived vulnerability and conspiracy beliefs. Although vulnerability is often assumed to increase motivation for self-protection, in highly uncertain and polarized contexts, it may also heighten psychological discomfort and motivate individuals to seek explanations that reduce ambiguity or restore a sense of meaning. Conspiracy beliefs may serve this function by offering seemingly coherent explanations for threatening events, particularly among individuals who already distrust institutions or rely on informal information channels. This pattern may also be understood through the lens of healthism, which emphasizes personal responsibility for health while often coexisting with skepticism toward medical and governmental institutions. From this perspective, feeling more vulnerable may not uniformly strengthen guideline adherence; instead, for some individuals, it may increase susceptibility to alternative explanations of the threat when official guidance is not perceived as trustworthy or effective [44].

## Implications

Together, these findings carry important implications for public health practice. First, they underscore the necessity of promoting formal sources of information. Government agencies, public health institutions, and established media remain critical in shaping accurate perceptions of severity and reducing susceptibility to conspiracy narratives. However, the results also caution that reliance on informal sources is not trivial. Social media, blogs, and peer networks are powerful forces that can reduce perceived severity, increase vulnerability to misinformation, and amplify ideological resistance. A successful communication strategy must therefore operate on both fronts: strengthening formal channels while actively addressing the content and influence of informal channels. Public health authorities cannot afford to cede the informal information space; rather, they must engage with it by disseminating accurate content through the very platforms where misinformation spreads most rapidly.

Second, the mediation findings highlight the mechanisms through which information exerts its influence. For mask-wearing, emphasizing the severity of disease outcomes remains central. Communication strategies that clearly convey the health risks of infection and the protective efficacy of masks can reinforce severity appraisals and, in turn, increase compliance. For vaccination, however, the challenge is more ideological. Uptake is shaped not only by risk perception but also by political identity and the alignment of messages with partisan frames. Communication strategies should therefore avoid a one-size-fits-all approach and instead tailor messages that resonate across ideological divides. For example, appeals to collective responsibility may be effective among liberals, while appeals to protecting family or economic stability may be more persuasive among conservatives.

Third, the observed clustering of behaviors suggests that interventions should be integrated rather than piecemeal. Encouraging compliance with one guideline may spill over into compliance with others. This finding supports the design of holistic campaigns that present preventive measures as mutually reinforcing components of a unified strategy, rather than as isolated behaviors.

## Limitations

Several limitations should be acknowledged. The study relied on self-reported data in a single way, which may have increased shared method variance and inflated some associations. In addition, the cross-sectional design prevents conclusions about causality or temporal ordering. Although the proposed model specifies pathways from information sources to beliefs and from beliefs to behaviors, longitudinal research is necessary to evaluate these directional assumptions more rigorously. The timing of data collection also warrants consideration. Because the data were collected in January 2023, participants’ responses were likely shaped by the particular public health, political, and informational environment of that period. Accordingly, the findings should be interpreted within that temporal context, and caution is needed in generalizing them to other phases of the pandemic or to different health crises. Moreover, the use of MTurk may limit representativeness, as online panel samples may not fully reflect the broader U.S. population, especially with respect to demographic diversity, health information access, and media use patterns. The model refinement strategy also resulted in the final model, which may reflect some degree of MTurk sample-specific optimization. In addition, the present study did not examine socioeconomic inequality as a central explanatory focus. Future research should investigate socioeconomic characteristics and cross-validate the model in more independent samples to determine whether the revised structure is stable and theoretically robust.

Another limitation concerns the distinction between formal and informal information sources. Although this categorization was useful for the purposes of analysis, contemporary information environments are often more blended than this dichotomy suggests. Official institutions frequently communicate through social media, and informal networks may also circulate messages originating from institutional sources. Therefore, these categories should be interpreted as broad operational groupings rather than as fully distinct information ecosystems. Future research would benefit from longitudinal and experimental designs, more diverse and representative samples, and data collection across multiple time points to better assess the stability and generalizability of these relationships.

## Conclusions

In conclusion, the present study provides compelling evidence that information sources are central drivers of protective behavior during a public health crisis. By demonstrating that reliance on formal versus informal channels sets in motion divergent cascades of risk appraisals, conspiracy beliefs, and political ideologies, the findings highlight the need to treat the information environment as a critical target of public health intervention. Future crises will inevitably be accompanied by competing narratives; preparing for these crises requires not only biomedical readiness but also robust strategies for information dissemination and misinformation management. Addressing both formal and informal channels, tailoring messages to diverse ideological audiences, and reinforcing the clustering of protective behaviors are essential steps for enhancing compliance and safeguarding public health.

## Supporting information

S1 File Dataset. (XLSX)
