# Supplementary material for: Why do people comply with health guidelines in a competing information environment?
Source: PLoS One. 2026 Jun 26;21(6):e0352421. doi: 10.1371/journal.pone.0352421 (PMC13308778; doi:10.1371/journal.pone.0352421)
Supplement: Attachment — Submitted filename: PLOS ONE Reviewer Comments.docx [file pone.0352421.s002.docx]

Dear Editors and Reviewers

Thank you for your time and for providing us with valuable feedback. We have revised our manuscript in response to the comments we received. Below is our point-by-point response to each comment:

1. Please ensure that your manuscript meets PLOS ONE's style requirements, including those for file naming. The PLOS ONE style templates can be found at

<https://journals.plos.org/plosone/s/file?id=wjVg/PLOSOne_formatting_sample_main_body.pdf> and

[https://journals.plos.org/plosone/s/file?id=ba62/PLOSOne_formatting_sample_title_authors_affiliations.pdf](https://track.editorialmanager.com/CL0/https:%2F%2Fjournals.plos.org%2Fplosone%2Fs%2Ffile%3Fid=ba62%2FPLOSOne_formatting_sample_title_authors_affiliations.pdf/1/010f019c30faf685-48261c52-7a37-4ca1-91f8-d57654e3c549-000000/X_BHv1myumCH3xYHRvuFqQ-PKvtdYAjr1HcVs7sE4Mo=249)

**Response**: Thank you for this feedback. We carefully reviewed the manuscript and revised it to conform to PLOS ONE formatting requirements.

2. In the online submission form, you indicated that [The data that support the findings of this study are available from the corresponding author upon reasonable request.].
All PLOS journals now require all data underlying the findings described in their manuscript to be freely available to other researchers, either 1. In a public repository, 2. Within the manuscript itself, or 3. Uploaded as supplementary information.

This policy applies to all data except where public deposition would breach compliance with the protocol approved by your research ethics board. If your data cannot be made publicly available for ethical or legal reasons (e.g., public availability would compromise patient privacy), please explain your reasons on resubmission and your exemption request will be escalated for approval.

**Response**: Thank you for this feedback. We have revised the Data Availability Statement to comply with PLOS ONE’s requirements. The data reported in this manuscript are now provided as supplementary information.

3. Please include your full ethics statement in the ‘Methods’ section of your manuscript file. In your statement, please include the full name of the IRB or ethics committee who approved or waived your study, as well as whether or not you obtained informed written or verbal consent. If consent was waived for your study, please include this information in your statement as well.

**Response**: Thank you for this feedback. We have included the full ethics statement in the ‘Methods’ section of our manuscript file.

Reviewer #1: This manuscript is a good job, the authors did excellent job but they need to do a minor formatting in their text , alignment and paragraphing.

Authors of this manuscript did a job except this minor issues of formatting .
I encourage the authors to conduct graphical or visual data visualization to enable the reader or stakeholders who are not good in numbers to understand the data generated.

**Response**: Thank you for your positive feedback. We have revised the manuscript to improve formatting to enhance overall readability and presentation. In addition, to make the findings more accessible to readers and stakeholders who may be less comfortable with numerical results, we clarified the visual presentation of the structural model and related results within the manuscript.

Reviewer #2: Your study on the influence of information sources on beliefs, risk perceptions, and protective behaviors during the COVID-19 pandemic in the U.S. population offers valuable insights into public health communication and compliance. The manuscript makes a meaningful contribution to public health and communication literature by elucidating the roles of information sources, conspiracy beliefs, and political ideology in shaping protective behaviors during the COVID-19 pandemic. Its use of PMT, rigorous SEM analysis, and practical implications are notable strengths. However, the study would benefit from deeper theoretical integration, greater methodological transparency, and more nuanced interpretation of unexpected findings. Addressing these concerns—particularly clarifying the role of conspiracy beliefs within PMT, justifying item removal, and tailoring practical implications—will enhance the manuscript’s quality and impact.

Specific revisions include:
1. Expanding the theoretical discussion to clarify how conspiracy beliefs and political ideology interact with PMT constructs (e.g., response efficacy).

**Response**: Thank you for this helpful comment. In the revised Introduction, we more explicitly integrated conspiracy beliefs and political orientation into the theoretical framework. Specifically, we clarified that these variables are treated as interpretive factors that shape threat appraisal and beliefs about the effectiveness of recommended protective actions.

2. Justifying item removal and discussing the MTurk sample’s representativeness, potentially with subgroup analyses.

**Response**: Thank you for this helpful comment. We clarified in the revised manuscript that the item removal process was conducted in our previous study during the scale development and refinement stage, rather than in the present study. Because the current manuscript is a secondary analysis using the previously refined measures, we did not repeat item-removal procedures here.

3. Deepening the interpretation of unexpected findings (e.g., vulnerability-conspiracy beliefs link) and tailoring practical implications to specific results.

**Response**: Thank you for this helpful comment. We revised the Discussion to provide a deeper interpretation of unexpected findings, particularly the positive association between perceived vulnerability and conspiracy beliefs. We also refined the practical implications so they are more closely tied to the specific findings of the study.

4. Addressing the temporal context (January 2023) in the discussion and limitations, and improving language clarity.

**Response:** Thank you for this helpful comment. We revised Limitations sections to more clearly acknowledge the temporal context of the study and to clarify how this context may have influenced participants’ attitudes, beliefs, and health-related behaviors.

Reviewer #3: This is a well written paper. However, title can be improved to better reflect the objectives of the paper. I have suggested few alternative titles. Similarly, justification and importance of the study can also be improved. I have written section-wise comments in the word file attached here. Hope this will help the authors to improve the quality.

Reviewer #4: The study assessed the sources of Health Guidelines Beliefs and Compliance, However, the following observations are made;
1. The study title is not that clear, there is therefore the need to modify the title to suit the content of the study. Something like “Determinants of Beliefs and Public Health Compliance. A Study of COVID-19 Guidelines Adherence, A Structural Equation Model”.
Also, authors must refrain from the use of flowery jargons. Eg propensity, gleaned, elucidate etc, it runs throughout the manuscript, such words are not academic. Authors must strive to use simple words that even primary school pupil reading the manuscript can understand.

**Response**: Thank you for this helpful suggestion. We revised the title to better align with the content of the manuscript. We also carefully edited the manuscript to simplify language and to improve readability throughout.

2. Authors need to structure the abstract well, no background was given, authors just started describing the objectives of the study. Authors must incorporate the background before zooming into the objectives.

**Response:** Thank you for this valuable feedback. We revised the abstract to include a clearer opening background before narrowing to the specific objectives of the study.

3. There’s a disconnect between the study design mentioned in the abstract and the one explained at the methods section. If cross-sectional was the design then authors must clearly explain what the cross-sectional study is, its strengths and weaknesses and the justification for its choice within the context of this study. Authors must strive to attain coherence across all segments of the paper.

**Response**: Thank you for this helpful comment. We revised the manuscript to ensure consistency in the description of the study design across the abstract and methods sections. We now clearly identify the study as cross-sectional throughout the manuscript and briefly explain what this design means in the context of the present study and its main strengths and limitations.

3. Authors indicated that ‘The study protocol received approval from the Institutional Review Board (IRB) prior to the commencement of the study’.
Authors must provide the name of the specific IRB and further provide approval number.

**Response**: Thank you for this helpful comment. We have revised the ethics statement to include the full name of the Institutional Review Board and the study approval number.

4. “The reliability of internal consistency was assessed through Cronbach's alpha in SPSS”. Authors must provide further details of the Cronbach Alpha, especially the value of the Cronbach Alpha that measured the internal consistency.

**Response**: Thank you for this helpful comment. Rather than presenting reliability information in a table, we now report the Cronbach’s alpha value for each measure directly after its description in the Measures section to make the internal consistency estimates easier to follow.

5. Some important elements that ought to accompany every standard research article is conspicuously missing in this manuscript. Authors must show us how they determined the sample size for this study, which formula was used ? at what CI?
Again, authors must clearly explain the specific sampling technique that was deployed and the rational for that technique.

**Response:** Thank you for this helpful comment. We have revised the Methods section to more clearly describe the sampling procedure and sample size determination.

6. The discussion segment of the study is poorly presented and it is highly defective. Discussion of results is not merely throwing more light on the results based on authors’ observations or understanding. However, the results must be contextualized in light of existing literature. The findings must either corroborate an earlier finding or differ from what is reported in literature. Hence, the discussion needs a serious overhaul ensure it conforms to standard scientific research article.
Authors must therefore get the relevant literatures to support the discussion of results.

**Response**: Thank you for this valuable comment. We substantially revised the Discussion section to better align the interpretation of our findings with the existing literature. We also incorporated additional relevant references throughout the Discussion to strengthen the scientific grounding of the manuscript.

7. Authors provided some implications for public health practice which is a typical recommendation, hence the heading should reflect as such. Authors have lumped it together as if it is part of the discussion.

**Response**: Thank you for this helpful comment. We have revised the manuscript to separate the practical recommendations from the main Discussion and to present them.

8. Again, there no heading called conclusion, so it is presumed that authors had no conclusions to show. This is a serious deficiency!.

**Response:** Thank you for this helpful comment. We have added a separate Conclusion section to the manuscript in order to clearly summarize the overall contribution of the study.

Reviewer #5: Dear Authors, thank you for providing me with the opportunity to review your work. While the research focus is novel, with its methodological approach in line with standard and acceptable scientific convention, i am of the opinion that the theoretical background of the paper still needs to be strengthened more. In this regard, i advise that a separate section should be created for discussing relevant theories, with such theories directly linked to the hypotheses that have been formulated.

**Response:** Thank you for this valuable comment. We revised the theoretical background section to more clearly present the relevant theories and to link them directly to the study hypotheses.

Reviewer #6: The manuscript addresses a timely and policy-relevant question concerning how different sources of health information shape beliefs, risk perceptions, and compliance with protective behaviors during the COVID-19 pandemic in the United States. By integrating information source use, conspiracy beliefs, perceived risk, and political ideology into a single structural equation model, the study offers an ambitious attempt to connect psychological and political mechanisms of health behavior. Its strengths include the explicit theoretical grounding in Protection Motivation Theory, the use of latent-variable SEM, and the examination of both mask-wearing and vaccination intentions. At the same time, several aspects of the conceptual framing, measurement strategy, and interpretation of the modeled relationships would benefit from clearer justification and more cautious inference to strengthen the manuscript’s contribution.

1. The manuscript conceptualizes “formal” and “informal” information sources as two distinct and opposing categories (“formal information includes government websites, national television, and reports from government leaders,” while “informal information incorporates sources like social media, friends, and family members”). While analytically convenient, this dichotomy risks oversimplifying contemporary information environments, where official actors often communicate through social media and informal sources may relay institutional messages. The authors should more explicitly acknowledge this conceptual overlap and clarify whether the constructs represent source credibility, institutional trust, or communication channels.

**Response:** Thank you for this insightful comment. In the revised manuscript, we clarified that these constructs are intended to reflect broad source categories as operationalized in the present study rather than mutually exclusive information ecosystems. We also added text to acknowledge the conceptual overlap between communication channels, source credibility, and institutional trust, and noted this issue as a limitation when interpreting the findings.

2. The theoretical framework draws primarily on Protection Motivation Theory, emphasizing perceived severity and vulnerability as mediators of protective behavior (“threat appraisal involves the assessment of the severity of the threat and one's vulnerability to it”). However, the integration of political ideology and conspiracy beliefs into this framework remains largely additive rather than theoretically synthesized. The manuscript would benefit from a clearer articulation of how ideological orientation conceptually fits within PMT, rather than functioning as an adjacent predictor.

**Response:** Thank you for this helpful comment. We strengthened the theoretical framing to clarify that political ideology and conspiracy beliefs are not treated as merely adjacent predictors, but as broader interpretive factors that shape appraisals and beliefs about the effectiveness of recommended protective actions.

3. Relatedly, the manuscript’s operationalization of perceived vulnerability and susceptibility could be strengthened by engaging with work that conceptualizes perceived threat as the combined appraisal of susceptibility and severity rather than as parallel indicators. Research grounded in threat appraisal frameworks demonstrates that treating perceived threat as a joint construct, often operationalized through the interaction or composite of perceived susceptibility and perceived severity, more closely reflects how individuals evaluate health risks and translate them into behavioral responses (https://www.frontiersin.org/journals/psychology/articles/10.3389/fpsyg.2024.1332697/full). Citing this work would provide a theoretically grounded rationale for conducting a sensitivity analysis using alternative threat operationalizations, allowing the authors to assess whether their findings regarding compliance intentions are robust to different, well-established measurement approaches.

**Response**: Thank you for this insightful comment. We revised the manuscript to better situate perceived susceptibility and perceived severity within the broader threat-appraisal literature. Specifically, we now acknowledge that these constructs are often treated as jointly constituting perceived threat, rather than only as parallel indicators, and we added the recommended citation to support this conceptualization.

4. The operationalization of political ideology relies on five items that explicitly reference liberal identification and Democratic Party alignment (“I think my political attitude is more associated with liberals”; “I generally favor the Democratic party over the Republican party”). This raises concerns about conflating ideological orientation with partisan identity. Given that the manuscript consistently refers to “political ideology,” greater conceptual precision is needed to clarify whether the latent construct represents ideology, partisanship, or a combination of both.

**Response:** Thank you for this insightful comment. We agree that the items used in this study capture not only ideological orientation but also elements of partisan identity. In response, we revised the manuscript to clarify that this latent construct reflects a broader political orientation that includes both ideological self-identification and party alignment, rather than a purely ideological measure.

5. The study relies on self-reported behavioral intentions rather than observed behaviors (“intention to wear masks” and “intention to get vaccinated”). While this is acknowledged implicitly, the interpretation occasionally treats intentions as direct indicators of compliance. The discussion would benefit from clearer differentiation between intention and behavior, particularly given evidence that intention-behavior gaps are common in health contexts.

**Response**: Thank you for this insightful comment. We revised the Discussion sections to more clearly distinguish behavioral intentions from observed behavior. We also acknowledged that intention–behavior gaps are common in health contexts and that this should be considered when interpreting the results.

6. Measurement decisions during scale refinement require more transparent justification. Several items were removed due to low loadings or redundancy (“FI6, PV1, and PI3 were excluded”; later, “II2, CT1, CT2, and CT7 were removed”). While such decisions are common in SEM, the manuscript would benefit from a clearer discussion of how item removal may have narrowed construct content, particularly for politically and socially sensitive constructs such as conspiracy beliefs.

**Response**: Thank you for this insightful comment. In the revised manuscript, we clarified that the item-removal decisions referenced in earlier drafts were part of a previously published scale-development/refinement study and are not the focus of the present analysis. To avoid duplicating procedures already reported elsewhere, we removed the detailed item-removal discussion from this manuscript.

7. The reported model modification strategy raises concerns about capitalizing on chance. The manuscript indicates that nonsignificant paths were removed to produce a better-fitting model (“paths deemed insignificant were removed from Model I, culminating in a revised model”). While improved fit indices are reported (CFI = 0.987; RMSEA = 0.071), greater caution is needed in interpreting the final model as theoretically definitive rather than statistically optimized for this specific sample.

**Response:** Thank you for this insightful comment. In the revised text, we clarify that the model refinement process was used to improve parsimony and fit within the current sample, and that the final model should be understood as an empirically refined representation rather than a conclusive theoretical model. We also acknowledge that removing nonsignificant paths may capitalize on sample-specific variation and that the final model should therefore be interpreted with appropriate caution and ideally examined in future replication studies.

8. Several reported standardized path coefficients are notably large, particularly between formal information and perceived severity (β = 0.77) and between formal information and vaccination intention (β = 0.45). The manuscript does not sufficiently discuss whether such strong effects are plausible given the cross-sectional design and shared-method measurement, nor whether multicollinearity among predictors may inflate estimates.

**Response:** Thank you for this insightful comment. We clarified the manuscript to interpret the larger standardized path coefficients more cautiously. Specifically, we noted that these paths should be understood as strong associations within a cross-sectional self-report design. We also acknowledged in the Limitations section that shared method variance may have contributed to the magnitude of some associations.

9. The role of conspiracy beliefs is presented as largely unidirectional, undermining compliance (“conspiracy theory beliefs had a negative association with intentions to get vaccinated”). However, the cross-sectional design does not allow determining whether conspiracy beliefs reduce compliance or whether resistance to compliance fosters conspiratorial thinking, nor whether the association primarily reflects stable between-person differences. This limitation should be more explicitly acknowledged when drawing implications for public health interventions. Longitudinal evidence using three-wave panel models indicates that conspiracy mentality and vaccine hesitancy can overlap strongly as stable dispositions, with weak or inconsistent within-person cross-lagged effects, and with trust-related pathways as an important mechanism (https://doi.org/10.1016/j.socscimed.2025.118770). Citing this work would help the manuscript position its cross-sectional findings more cautiously while clarifying what kinds of causal claims require panel or within-person designs.

**Response**: Thank you for this insightful comment. We revised the manuscript to interpret the association between conspiracy beliefs and vaccination intention more cautiously. We also incorporated the recommended longitudinal study to note that conspiracy mentality and vaccine hesitancy may overlap substantially as stable dispositions.

10. The sample is drawn from MTurk and is relatively highly educated (“48.0% bachelor’s degree; 20.8% master’s degree”), yet education is not incorporated into the structural model. Given the manuscript’s emphasis on information processing and belief formation, the absence of education or socioeconomic status from the analytical model represents a missed opportunity to assess structural inequalities in guideline compliance.

**Response**: Thank you for this insightful comment. We agree that education and socioeconomic status are important factors. However, the primary focus of the present study was to examine the proposed structural model linking information sources, risk perceptions, sociopolitical beliefs, and protective intentions. Accordingly, these variables were not included as focal predictors in the current analysis. We now acknowledge this as a limitation in the manuscript and note that future research should examine education and socioeconomic factors more directly in order to better understand their role in shaping these processes.

11. The manuscript reports high explained variance for key outcomes (“72.8% of the variance in intentions to get vaccinated”), which is notable for social-behavioral research. However, little discussion is devoted to whether this reflects substantive explanatory power or shared measurement variance among attitudinal constructs measured using the same Likert scale and survey context.

**Response**: Thank you for this important comment. We revised the Discussion and Limitations sections to more carefully interpret the high explained variance reported for key outcomes. We also acknowledged in the Limitations section that shared method variance may have contributed to the magnitude of some associations.

12. Although the manuscript frequently references trust and misinformation, institutional trust is not directly measured. Instead, trust is inferred through source preference and ideology (“formal sources associated with liberalism and compliance”). The authors should be more cautious in interpreting these pathways as evidence of trust, rather than preference alignment or identity-consistent information seeking (see point 9).

**Response**: Thank you for this insightful comment. We agree that institutional trust was not directly measured in the present study and therefore should not be inferred too strongly from source preference. In the revised manuscript, we now interpret these pathways more cautiously and avoid presenting them as direct evidence of trust. Instead, we clarify that the observed associations may also reflect preference alignment or identity-consistent information seeking toward particular information environments.

13. The manuscript’s interpretation of compliance and resistance would benefit from engaging with research on healthism, which conceptualizes health-related behavior as shaped by strong emphases on personal responsibility combined with skepticism toward medical and governmental institutions. Empirical work shows that individuals who stress self-control over health outcomes while distrusting institutional actors may actively seek information and appraise risks, yet still resist externally imposed guidelines, even when perceived severity is high (https://doi.org/10.3390/su15076107). Incorporating this perspective would help clarify why exposure to formal information sources and heightened risk perception do not uniformly translate into compliance, and would allow the manuscript to situate conspiracy beliefs and ideological resistance within broader cultural orientations toward health governance rather than treating them solely as information deficits.

**Response:** Thank you for this insightful comment. We revised the Discussion to incorporate the concept of healthism and cited the recommended study. In doing so, we clarified that resistance to health guidelines may not stem solely from misinformation or lack of awareness; rather, individuals actively monitor risk and seek health information while still rejecting externally imposed guidance when health is viewed as a matter of personal responsibility or governmental institutions are met with skepticism.

14. Finally, while the discussion draws strong policy implications regarding communication strategies (“clear and consistent messaging from trusted authorities can help rectify misinformation”), these recommendations extend beyond what the empirical design can directly support. The manuscript would benefit from more clearly distinguishing empirically demonstrated associations from normative or strategic recommendations.
The manuscript addresses an important and policy-relevant question concerning the determinants of compliance with public health guidelines and presents careful and technically competent empirical analyses. However, its contribution would be strengthened by deeper theoretical integration between information sources, ideology, and protection motivation, clearer and more cautious interpretation of the modeled relationships, and a more explicit discussion of measurement choices and design limitations inherent in the cross-sectional SEM approach.

**Response:** Thank you for this thoughtful and constructive summary. We revised the manuscript to more carefully align our interpretations and policy implications with what can be supported by the present cross-sectional SEM design. We also strengthened the theoretical framing to more explicitly connect information sources, ideology, and protection motivation, and expanded the discussion of design limitations, including the constraints of cross-sectional data and the implications of model trimming and sample characteristics for interpretation and generalizability.
