# Supplementary material for: Why do people comply with health guidelines in a competing information environment?
Source: PLoS One. 2026 Jun 26;21(6):e0352421. doi: 10.1371/journal.pone.0352421 (PMC13308778; doi:10.1371/journal.pone.0352421)
Supplement: Attachment — Submitted filename: Response to reviewer comments v2.docx [file pone.0352421.s003.docx]

Please review your reference list to ensure that it is complete and correct. If you have cited papers that have been retracted, please include the rationale for doing so in the manuscript text, or remove these references and replace them with relevant current references. Any changes to the reference list should be mentioned in the rebuttal letter that accompanies your revised manuscript. If you need to cite a retracted article, indicate the article’s retracted status in the References list and also include a citation and full reference for the retraction notice.

Response: Thank you for the feedback. We carefully reviewed our reference list and ensure that it is complete and correct.

Reviewer #1: The author needs to improve the formatting by ensuring proper use of capital and lowercase letters, commas, and colons. Please work on the overall formatting and writing style to enhance clarity and readability. In addition, strengthen the data analysis to make it more robust and comprehensive. The title should also be revised to make it more professional, smart, and captivating.

**Response**: Thank you for this helpful suggestion. We carefully reviewed the manuscript to improve formatting. We also revised the writing throughout the manuscript to enhance clarity and readability. In addition, we revised the title to make it more professional, smart, and captivating. We agree that the data analysis should be presented in a clear and comprehensive manner. However, we believe that the current analytic approach is appropriate and sufficiently comprehensive for the aims of the study. The manuscript uses structural equation modeling to evaluate the hypothesized relationships, reports model comparison using established fit indices, presents standardized path coefficients, and includes explained variance for the endogenous variables. Given that the purpose of the study was to test the proposed structural pathways rather than to conduct additional sensitivity analyses, we retained the current analytic framework to maintain a focused and theoretically aligned presentation of the findings.

Reviewer #4: Authors have duly incorporated my recommendations which has significantly improved the manuscript It is now suitable for publication

Reviewer #7: The authors have adequately addressed the concerns raised in the previous review round. The manuscript has been substantially improved, particularly in terms of theoretical integration, clarification of limitations, and interpretation of the SEM findings. The revisions strengthened the discussion of cross-sectional design limitations, information-source categorization, and ideological pathways influencing health behaviors. The manuscript is technically sound, clearly written, and suitable for publication

**Response**: Thank you for the feedback. We sincerely appreciate the reviewers’ careful evaluation of the revised manuscript and are pleased that the revisions have addressed the concerns raised in the previous review round.

Reviewer #6: The paper is improved and publishable in my opinion. I would suggest the data availability statement to be included in one of the segments of the manuscript (e.g., at the end of the manuscript).

**Response**: Thank you for this helpful suggestion. We added the data availability statement in Results section.
