# Supplementary material for: Why do people comply with health guidelines in a competing information environment?
Source: PLoS One. 2026 Jun 26;21(6):e0352421. doi: 10.1371/journal.pone.0352421 (PMC13308778; doi:10.1371/journal.pone.0352421)
Supplement: Peer review history

# Peer review: Decision Letter 0

**DOI:** 10.1371/journal.pone.0352421.r001

**Authors:** Chao Gu

5 Feb 2026

PONE-D-24-51218

Sources of Health Guidelines Beliefs and Compliances: A Structural Equation Model

PLOS One

Dear Dr. Yu,

Thank you for submitting your manuscript to PLOS ONE. After careful consideration, we feel that it has merit but does not fully meet PLOS ONE’s publication criteria as it currently stands. Therefore, we invite you to submit a revised version of the manuscript that addresses the points raised during the review process.

**ACADEMIC EDITOR:**   Please make substantive revisions in accordance with the reviewers’ comments.

Please submit your revised manuscript by Mar 22 2026 11:59PM. If you will need more time than this to complete your revisions, please reply to this message or contact the journal office at plosone@plos.org. When you're ready to submit your revision, log on to https://www.editorialmanager.com/pone/ and select the 'Submissions Needing Revision' folder to locate your manuscript file.

Please include the following items when submitting your revised manuscript:

- A letter that responds to each point raised by the academic editor and reviewer(s). You should upload this letter as a separate file labeled 'Response to Reviewers'.
- A marked-up copy of your manuscript that highlights changes made to the original version. You should upload this as a separate file labeled 'Revised Manuscript with Track Changes'.
- An unmarked version of your revised paper without tracked changes. You should upload this as a separate file labeled 'Manuscript'.

If you would like to make changes to your financial disclosure, please include your updated statement in your cover letter. Guidelines for resubmitting your figure files are available below the reviewer comments at the end of this letter.

If applicable, we recommend that you deposit your laboratory protocols in protocols.io to enhance the reproducibility of your results. Protocols.io assigns your protocol its own identifier (DOI) so that it can be cited independently in the future. For instructions see: https://journals.plos.org/plosone/s/submission-guidelines#loc-laboratory-protocols. Additionally, PLOS ONE offers an option for publishing peer-reviewed Lab Protocol articles, which describe protocols hosted on protocols.io. Read more information on sharing protocols at https://plos.org/protocols?utm_medium=editorial-email&utm_source=authorletters&utm_campaign=protocols.

We look forward to receiving your revised manuscript.

Kind regards,

Chao Gu

Academic Editor

PLOS One

Journal Requirements:

When submitting your revision, we need you to address these additional requirements.

1. Please ensure that your manuscript meets PLOS ONE's style requirements, including those for file naming. The PLOS ONE style templates can be found at

https://journals.plos.org/plosone/s/file?id=wjVg/PLOSOne_formatting_sample_main_body.pdf and

https://journals.plos.org/plosone/s/file?id=ba62/PLOSOne_formatting_sample_title_authors_affiliations.pdf

2. In the online submission form, you indicated that [The data that support the findings of this study are available from the corresponding author upon reasonable request.].

All PLOS journals now require all data underlying the findings described in their manuscript to be freely available to other researchers, either 1. In a public repository, 2. Within the manuscript itself, or 3. Uploaded as supplementary information.

This policy applies to all data except where public deposition would breach compliance with the protocol approved by your research ethics board. If your data cannot be made publicly available for ethical or legal reasons (e.g., public availability would compromise patient privacy), please explain your reasons on resubmission and your exemption request will be escalated for approval.

3. Please include your full ethics statement in the ‘Methods’ section of your manuscript file. In your statement, please include the full name of the IRB or ethics committee who approved or waived your study, as well as whether or not you obtained informed written or verbal consent. If consent was waived for your study, please include this information in your statement as well.

4. If the reviewer comments include a recommendation to cite specific previously published works, please review and evaluate these publications to determine whether they are relevant and should be cited. There is no requirement to cite these works unless the editor has indicated otherwise.

[Note: HTML markup is below. Please do not edit.]

Reviewers' comments:

Reviewer's Responses to Questions

**Comments to the Author**

1. Is the manuscript technically sound, and do the data support the conclusions?

The manuscript must describe a technically sound piece of scientific research with data that supports the conclusions. Experiments must have been conducted rigorously, with appropriate controls, replication, and sample sizes. The conclusions must be drawn appropriately based on the data presented.

Reviewer #1: Yes

Reviewer #2: Yes

Reviewer #3: Yes

Reviewer #4: Partly

Reviewer #5: Yes

Reviewer #6: Partly

**********

2. Has the statistical analysis been performed appropriately and rigorously?

Reviewer #1: Yes

Reviewer #2: Yes

Reviewer #3: Yes

Reviewer #4: Yes

Reviewer #5: Yes

Reviewer #6: Yes

**********

3. Have the authors made all data underlying the findings in their manuscript fully available?

The PLOS Data policy requires authors to make all data underlying the findings described in their manuscript fully available without restriction, with rare exception (please refer to the Data Availability Statement in the manuscript PDF file). The data should be provided as part of the manuscript or its supporting information, or deposited to a public repository. For example, in addition to summary statistics, the data points behind means, medians and variance measures should be available. If there are restrictions on publicly sharing data—e.g. participant privacy or use of data from a third party—those must be specified.

Reviewer #1: Yes

Reviewer #2: Yes

Reviewer #3: No

Reviewer #4: Yes

Reviewer #5: No

Reviewer #6: No

**********

4. Is the manuscript presented in an intelligible fashion and written in standard English?

PLOS ONE does not copyedit accepted manuscripts, so the language in submitted articles must be clear, correct, and unambiguous. Any typographical or grammatical errors should be corrected at revision, so please note any specific errors here.

Reviewer #1: Yes

Reviewer #2: Yes

Reviewer #3: Yes

Reviewer #4: Yes

Reviewer #5: Yes

Reviewer #6: Yes

**********

5. Review Comments to the Author

Please use the space provided to explain your answers to the questions above. You may also include additional comments for the author, including concerns about dual publication, research ethics, or publication ethics. (Please upload your review as an attachment if it exceeds 20,000 characters)

Reviewer #1: This manuscript is a good job , the authors did excellent job but they need to do a minor formatting in their text , alignment and paragraphing .Authors of this manuscript did a job except this minor issues of formatting .

I encourage the authors to conduct graphical or visual data visualization to enable the reader or stakeholders who are not good in numbers to understand the data generated .

Reviewer #2: Your study on the influence of information sources on beliefs, risk perceptions, and protective behaviors during the COVID-19 pandemic in the U.S. population offers valuable insights into public health communication and compliance. The manuscript makes a meaningful contribution to public health and communication literature by elucidating the roles of information sources, conspiracy beliefs, and political ideology in shaping protective behaviors during the COVID-19 pandemic. Its use of PMT, rigorous SEM analysis, and practical implications are notable strengths. However, the study would benefit from deeper theoretical integration, greater methodological transparency, and more nuanced interpretation of unexpected findings. Addressing these concerns—particularly clarifying the role of conspiracy beliefs within PMT, justifying item removal, and tailoring practical implications—will enhance the manuscript’s quality and impact.

Specific revisions include:

1. Expanding the theoretical discussion to clarify how conspiracy beliefs and political ideology interact with PMT constructs (e.g., response efficacy).

2. Justifying item removal and discussing the MTurk sample’s representativeness, potentially with subgroup analyses.

3. Deepening the interpretation of unexpected findings (e.g., vulnerability-conspiracy beliefs link) and tailoring practical implications to specific results.

4. Addressing the temporal context (January 2023) in the discussion and limitations, and improving language clarity.

Reviewer #3: This is a well written paper. However, title can be improved to better reflect the objectives of the paper. I have suggested few alternative titles. Similarly, justification and importance of the study can also be improved. I have written section-wise comments in the word file attached here. Hope this will help the authors to improve the quality.

Reviewer #4: The study assessed the sources of Health Guidelines Beliefs and Compliance, However, the following observations are made;

1. The study title is not that clear, there is therefore the need to modify the title to suit the content of the study. Something like “Determinants of Beliefs and Public Health Compliance. A Study of COVID-19 Guidelines Adherence, A Structural Equation Model”.

Also, authors must refrain from the use of flowery jargons. Eg propensity, gleaned, elucidate etc, it runs throughout the manuscript, such words are not academic. Authors must strive to use simple words that even primary school pupil reading the manuscript can understand.

2. Authors need to structure the abstract well, no background was given, authors just started describing the objectives of the study. Authors must incorporate the background before zooming into the objectives.

3. There’s a disconnect between the study design mentioned in the abstract and the one explained at the methods section. If cross-sectional was the design then authors must clearly explain what the cross-sectional study is, its strengths and weaknesses and the justification for its choice within the context of this study. Authors must strive to attain coherence across all segments of the paper.

3. Authors indicated that ‘The study protocol received approval from the Institutional Review Board (IRB) prior to the commencement of the study’.

Authors must provide the name of the specific IRB and further provide approval number.

4. “The reliability of internal consistency was assessed through Cronbach's alpha in SPSS”. Authors must provide further details of the Cronbach Alpha, especially the value of the Cronbach Alpha that measured the internal consistency.

5. Some important elements that ought to accompany every standard research article is conspicuously missing in this manuscript. Authors must show us how they determined the sample size for this study, which formula was used ? at what CI?

Again, authors must clearly explain the specific sampling technique that was deployed and the rational for that technique.

6. The discussion segment of the study is poorly presented and it is highly defective. Discussion of results is not merely throwing more light on the results based on authors’ observations or understanding. However, the results must be contextualized in light of existing literature. The findings must either corroborate an earlier finding or differ from what is reported in literature. Hence, the discussion needs a serious overhaul ensure it conforms to standard scientific research article.

Authors must therefore get the relevant literatures to support the discussion of results.

7. Authors provided some implications for public health practice which is a typical recommendation, hence the heading should reflect as such. Authors have lumped it together as if it is part of the discussion.

8. Again, there no heading called conclusion, so it is presumed that authors had no conclusions to show. This is a serious deficiency!.

9. Until the above anomalies are rectified, I cannot accept the findings emanating from this study.

Reviewer #5: Dear Authors, thank you for providing me with the opportunity to review your work. While the research focus is novel, with its methodological approach in line with standard and acceptable scientific convention, i am of the opinion that the theoretical background of the paper still needs to be strengthened more. In this regard, i advise that a separate section should be created for discussing relevant theories, with such theories directly linked to the hypotheses that have been formulated.

Reviewer #6: The manuscript addresses a timely and policy-relevant question concerning how different sources of health information shape beliefs, risk perceptions, and compliance with protective behaviors during the COVID-19 pandemic in the United States. By integrating information source use, conspiracy beliefs, perceived risk, and political ideology into a single structural equation model, the study offers an ambitious attempt to connect psychological and political mechanisms of health behavior. Its strengths include the explicit theoretical grounding in Protection Motivation Theory, the use of latent-variable SEM, and the examination of both mask-wearing and vaccination intentions. At the same time, several aspects of the conceptual framing, measurement strategy, and interpretation of the modeled relationships would benefit from clearer justification and more cautious inference to strengthen the manuscript’s contribution.

1. The manuscript conceptualizes “formal” and “informal” information sources as two distinct and opposing categories (“formal information includes government websites, national television, and reports from government leaders,” while “informal information incorporates sources like social media, friends, and family members”). While analytically convenient, this dichotomy risks oversimplifying contemporary information environments, where official actors often communicate through social media and informal sources may relay institutional messages. The authors should more explicitly acknowledge this conceptual overlap and clarify whether the constructs represent source credibility, institutional trust, or communication channels.

2. The theoretical framework draws primarily on Protection Motivation Theory, emphasizing perceived severity and vulnerability as mediators of protective behavior (“threat appraisal involves the assessment of the severity of the threat and one's vulnerability to it”). However, the integration of political ideology and conspiracy beliefs into this framework remains largely additive rather than theoretically synthesized. The manuscript would benefit from a clearer articulation of how ideological orientation conceptually fits within PMT, rather than functioning as an adjacent predictor.

3. Relatedly, the manuscript’s operationalization of perceived vulnerability and susceptibility could be strengthened by engaging with work that conceptualizes perceived threat as the combined appraisal of susceptibility and severity rather than as parallel indicators. Research grounded in threat appraisal frameworks demonstrates that treating perceived threat as a joint construct, often operationalized through the interaction or composite of perceived susceptibility and perceived severity, more closely reflects how individuals evaluate health risks and translate them into behavioral responses (https://www.frontiersin.org/journals/psychology/articles/10.3389/fpsyg.2024.1332697/full). Citing this work would provide a theoretically grounded rationale for conducting a sensitivity analysis using alternative threat operationalizations, allowing the authors to assess whether their findings regarding compliance intentions are robust to different, well-established measurement approaches.

4. The operationalization of political ideology relies on five items that explicitly reference liberal identification and Democratic Party alignment (“I think my political attitude is more associated with liberals”; “I generally favor the Democratic party over the Republican party”). This raises concerns about conflating ideological orientation with partisan identity. Given that the manuscript consistently refers to “political ideology,” greater conceptual precision is needed to clarify whether the latent construct represents ideology, partisanship, or a combination of both.

5. The study relies on self-reported behavioral intentions rather than observed behaviors (“intention to wear masks” and “intention to get vaccinated”). While this is acknowledged implicitly, the interpretation occasionally treats intentions as direct indicators of compliance. The discussion would benefit from clearer differentiation between intention and behavior, particularly given evidence that intention-behavior gaps are common in health contexts.

6. Measurement decisions during scale refinement require more transparent justification. Several items were removed due to low loadings or redundancy (“FI6, PV1, and PI3 were excluded”; later, “II2, CT1, CT2, and CT7 were removed”). While such decisions are common in SEM, the manuscript would benefit from a clearer discussion of how item removal may have narrowed construct content, particularly for politically and socially sensitive constructs such as conspiracy beliefs.

7. The reported model modification strategy raises concerns about capitalizing on chance. The manuscript indicates that nonsignificant paths were removed to produce a better-fitting model (“paths deemed insignificant were removed from Model I, culminating in a revised model”). While improved fit indices are reported (CFI = 0.987; RMSEA = 0.071), greater caution is needed in interpreting the final model as theoretically definitive rather than statistically optimized for this specific sample.

8. Several reported standardized path coefficients are notably large, particularly between formal information and perceived severity (β = 0.77) and between formal information and vaccination intention (β = 0.45). The manuscript does not sufficiently discuss whether such strong effects are plausible given the cross-sectional design and shared-method measurement, nor whether multicollinearity among predictors may inflate estimates.

9. The role of conspiracy beliefs is presented as largely unidirectional, undermining compliance (“conspiracy theory beliefs had a negative association with intentions to get vaccinated”). However, the cross-sectional design does not allow determining whether conspiracy beliefs reduce compliance or whether resistance to compliance fosters conspiratorial thinking, nor whether the association primarily reflects stable between-person differences. This limitation should be more explicitly acknowledged when drawing implications for public health interventions. Longitudinal evidence using three-wave panel models indicates that conspiracy mentality and vaccine hesitancy can overlap strongly as stable dispositions, with weak or inconsistent within-person cross-lagged effects, and with trust-related pathways as an important mechanism (https://doi.org/10.1016/j.socscimed.2025.118770). Citing this work would help the manuscript position its cross-sectional findings more cautiously while clarifying what kinds of causal claims require panel or within-person designs.

10. The sample is drawn from MTurk and is relatively highly educated (“48.0% bachelor’s degree; 20.8% master’s degree”), yet education is not incorporated into the structural model. Given the manuscript’s emphasis on information processing and belief formation, the absence of education or socioeconomic status from the analytical model represents a missed opportunity to assess structural inequalities in guideline compliance.

11. The manuscript reports high explained variance for key outcomes (“72.8% of the variance in intentions to get vaccinated”), which is notable for social-behavioral research. However, little discussion is devoted to whether this reflects substantive explanatory power or shared measurement variance among attitudinal constructs measured using the same Likert scale and survey context.

12. Although the manuscript frequently references trust and misinformation, institutional trust is not directly measured. Instead, trust is inferred through source preference and ideology (“formal sources associated with liberalism and compliance”). The authors should be more cautious in interpreting these pathways as evidence of trust, rather than preference alignment or identity-consistent information seeking (see point 9).

13. The manuscript’s interpretation of compliance and resistance would benefit from engaging with research on healthism, which conceptualizes health-related behavior as shaped by strong emphases on personal responsibility combined with skepticism toward medical and governmental institutions. Empirical work shows that individuals who stress self-control over health outcomes while distrusting institutional actors may actively seek information and appraise risks, yet still resist externally imposed guidelines, even when perceived severity is high (https://doi.org/10.3390/su15076107). Incorporating this perspective would help clarify why exposure to formal information sources and heightened risk perception do not uniformly translate into compliance, and would allow the manuscript to situate conspiracy beliefs and ideological resistance within broader cultural orientations toward health governance rather than treating them solely as information deficits.

14. Finally, while the discussion draws strong policy implications regarding communication strategies (“clear and consistent messaging from trusted authorities can help rectify misinformation”), these recommendations extend beyond what the empirical design can directly support. The manuscript would benefit from more clearly distinguishing empirically demonstrated associations from normative or strategic recommendations.

The manuscript addresses an important and policy-relevant question concerning the determinants of compliance with public health guidelines and presents careful and technically competent empirical analyses. However, its contribution would be strengthened by deeper theoretical integration between information sources, ideology, and protection motivation, clearer and more cautious interpretation of the modeled relationships, and a more explicit discussion of measurement choices and design limitations inherent in the cross-sectional SEM approach. Addressing the issues outlined above would substantially strengthen the manuscript’s contribution to research on health communication, risk perception, and public health compliance. I suggest a major revision.

**********

6. PLOS authors have the option to publish the peer review history of their article (what does this mean?). If published, this will include your full peer review and any attached files.

If you choose “no”, your identity will remain anonymous but your review may still be made public.

**Do you want your identity to be public for this peer review?**   For information about this choice, including consent withdrawal, please see our Privacy Policy.

Reviewer #1: **Yes:**   Daniel Akubueze

Reviewer #2: No

Reviewer #3: No

Reviewer #4: **Yes:**   ANTWI JOSEPH BARIMAH

Reviewer #5: **Yes:**   Adewale Adekiya

Reviewer #6: No

**********

[NOTE: If reviewer comments were submitted as an attachment file, they will be attached to this email and accessible via the submission site. Please log into your account, locate the manuscript record, and check for the action link "View Attachments". If this link does not appear, there are no attachment files.]

To ensure your figures meet our technical requirements, please review our figure guidelines: https://journals.plos.org/plosone/s/figures

You may also use PLOS’s free figure tool, NAAS, to help you prepare publication quality figures: https://journals.plos.org/plosone/s/figures#loc-tools-for-figure-preparation.

NAAS will assess whether your figures meet our technical requirements by comparing each figure against our figure specifications.

# Author comment: Author response to Decision Letter 1

**DOI:** 10.1371/journal.pone.0352421.r002

20 Mar 2026

Dear Editors and Reviewers

Thank you for your time and for providing us with valuable feedback. We have revised our manuscript in response to the comments we received. Below is our point-by-point response to each comment:

1. Please ensure that your manuscript meets PLOS ONE's style requirements, including those for file naming. The PLOS ONE style templates can be found at

https://journals.plos.org/plosone/s/file?id=wjVg/PLOSOne_formatting_sample_main_body.pdf and

https://journals.plos.org/plosone/s/file?id=ba62/PLOSOne_formatting_sample_title_authors_affiliations.pdf

Response: Thank you for this feedback. We carefully reviewed the manuscript and revised it to conform to PLOS ONE formatting requirements.

2. In the online submission form, you indicated that [The data that support the findings of this study are available from the corresponding author upon reasonable request.].

All PLOS journals now require all data underlying the findings described in their manuscript to be freely available to other researchers, either 1. In a public repository, 2. Within the manuscript itself, or 3. Uploaded as supplementary information.

This policy applies to all data except where public deposition would breach compliance with the protocol approved by your research ethics board. If your data cannot be made publicly available for ethical or legal reasons (e.g., public availability would compromise patient privacy), please explain your reasons on resubmission and your exemption request will be escalated for approval.

Response: Thank you for this feedback. We have revised the Data Availability Statement to comply with PLOS ONE’s requirements. The data reported in this manuscript are now provided as supplementary information.

3. Please include your full ethics statement in the ‘Methods’ section of your manuscript file. In your statement, please include the full name of the IRB or ethics committee who approved or waived your study, as well as whether or not you obtained informed written or verbal consent. If consent was waived for your study, please include this information in your statement as well.

Response: Thank you for this feedback. We have included the full ethics statement in the ‘Methods’ section of our manuscript file.

Reviewer #1: This manuscript is a good job, the authors did excellent job but they need to do a minor formatting in their text , alignment and paragraphing.

Authors of this manuscript did a job except this minor issues of formatting .

I encourage the authors to conduct graphical or visual data visualization to enable the reader or stakeholders who are not good in numbers to understand the data generated.

Response: Thank you for your positive feedback. We have revised the manuscript to improve formatting to enhance overall readability and presentation. In addition, to make the findings more accessible to readers and stakeholders who may be less comfortable with numerical results, we clarified the visual presentation of the structural model and related results within the manuscript.

Reviewer #2: Your study on the influence of information sources on beliefs, risk perceptions, and protective behaviors during the COVID-19 pandemic in the U.S. population offers valuable insights into public health communication and compliance. The manuscript makes a meaningful contribution to public health and communication literature by elucidating the roles of information sources, conspiracy beliefs, and political ideology in shaping protective behaviors during the COVID-19 pandemic. Its use of PMT, rigorous SEM analysis, and practical implications are notable strengths. However, the study would benefit from deeper theoretical integration, greater methodological transparency, and more nuanced interpretation of unexpected findings. Addressing these concerns—particularly clarifying the role of conspiracy beliefs within PMT, justifying item removal, and tailoring practical implications—will enhance the manuscript’s quality and impact.

Specific revisions include:

1. Expanding the theoretical discussion to clarify how conspiracy beliefs and political ideology interact with PMT constructs (e.g., response efficacy).

Response: Thank you for this helpful comment. In the revised Introduction, we more explicitly integrated conspiracy beliefs and political orientation into the theoretical framework. Specifically, we clarified that these variables are treated as interpretive factors that shape threat appraisal and beliefs about the effectiveness of recommended protective actions.

2. Justifying item removal and discussing the MTurk sample’s representativeness, potentially with subgroup analyses.

Response: Thank you for this helpful comment. We clarified in the revised manuscript that the item removal process was conducted in our previous study during the scale development and refinement stage, rather than in the present study. Because the current manuscript is a secondary analysis using the previously refined measures, we did not repeat item-removal procedures here.

3. Deepening the interpretation of unexpected findings (e.g., vulnerability-conspiracy beliefs link) and tailoring practical implications to specific results.

Response: Thank you for this helpful comment. We revised the Discussion to provide a deeper interpretation of unexpected findings, particularly the positive association between perceived vulnerability and conspiracy beliefs. We also refined the practical implications so they are more closely tied to the specific findings of the study.

4. Addressing the temporal context (January 2023) in the discussion and limitations, and improving language clarity.

Response: Thank you for this helpful comment. We revised Limitations sections to more clearly acknowledge the temporal context of the study and to clarify how this context may have influenced participants’ attitudes, beliefs, and health-related behaviors.

Reviewer #3: This is a well written paper. However, title can be improved to better reflect the objectives of the paper. I have suggested few alternative titles. Similarly, justification and importance of the study can also be improved. I have written section-wise comments in the word file attached here. Hope this will help the authors to improve the quality.

Reviewer #4: The study assessed the sources of Health Guidelines Beliefs and Compliance, However, the following observations are made;

1. The study title is not that clear, there is therefore the need to modify the title to suit the content of the study. Something like “Determinants of Beliefs and Public Health Compliance. A Study of COVID-19 Guidelines Adherence, A Structural Equation Model”.

Also, authors must refrain from the use of flowery jargons. Eg propensity, gleaned, elucidate etc, it runs throughout the manuscript, such words are not academic. Authors must strive to use simple words that even primary school pupil reading the manuscript can understand.

Response: Thank you for this helpful suggestion. We revised the title to better align with the content of the manuscript. We also carefully edited the manuscript to simplify language and to improve readability throughout.

2. Authors need to structure the abstract well, no background was given, authors just started describing the objectives of the study. Authors must incorporate the background before zooming into the objectives.

Response: Thank you for this valuable feedback. We revised the abstract to include a clearer opening background before narrowing to the specific objectives of the study.

3. There’s a disconnect between the study design mentioned in the abstract and the one explained at the methods section. If cross-sectional was the design then authors must clearly explain what the cross-sectional study is, its strengths and weaknesses and the justification for its choice within the context of this study. Authors must strive to attain coherence across all segments of the paper.

Response: Thank you for this helpful comment. We revised the manuscript to ensure consistency in the description of the study design across the abstract and methods sections. We now clearly identify the study as cross-sectional throughout the manuscript and briefly explain what this design means in the context of the present study and its main strengths and limitations.

3. Authors indicated that ‘The study protocol received approval from the Institutional Review Board (IRB) prior to the commencement of the study’.

Authors must provide the name of the specific IRB and further provide approval number.

Response: Thank you for this helpful comment. We have revised the ethics statement to include the full name of the Institutional Review Board and the study approval number.

4. “The reliability of internal consistency was assessed through Cronbach's alpha in SPSS”. Authors must provide further details of the Cronbach Alpha, especially the value of the Cronbach Alpha that measured the internal consistency.

Response: Thank you for this helpful comment. Rather than presenting reliability information in a table, we now report the Cronbach’s alpha value for each measure directly after its description in the Measures section to make the internal consistency estimates easier to follow.

5. Some important elements that ought to accompany every standard research article is conspicuously missing in this manuscript. Authors must show us how they determined the sample size for this study, which formula was used ? at what CI?

Again, authors must clearly explain the specific sampling technique that was deployed and the rational for that technique.

Response: Thank you for this helpful comment. We have revised the Methods section to more clearly describe the sampling procedure and sample size determination.

6. The discussion segment of the study is poorly presented and it is highly defective. Discussion of results is not merely throwing more light on the results based on authors’ observations or understanding. However, the results must be contextualized in light of existing literature. The findings must either corroborate an earlier finding or differ from what is reported in literature. Hence, the discussion needs a serious overhaul ensure it conforms to standard scientific research article.

Authors must therefore get the relevant literatures to support the discussion of results.

Response: Thank you for this valuable comment. We substantially revised the Discussion section to better align the interpretation of our findings with the existing literature. We also incorporated additional relevant references throughout the Discussion to strengthen the scientific grounding of the manuscript.

7. Authors provided some implications for public health practice which is a typical recommendation, hence the heading should reflect as such. Authors have lumped it together as if it is part of the discussion.

Response: Thank you for this helpful comment. We have revised the manuscript to separate the practical recommendations from the main Discussion and to present them.

8. Again, there no heading called conclusion, so it is presumed that authors had no conclusions to show. This is a serious deficiency!.

Response: Thank you for this helpful comment. We have added a separate Conclusion section to the manuscript in order to clearly summarize the overall contribution of the study.

Reviewer #5: Dear Authors, thank you for providing me with the opportunity to review your work. While the research focus is novel, with its methodological approach in line with standard and acceptable scientific convention, i am of the opinion that the theoretical background of the paper still needs to be strengthened more. In this regard, i advise that a separate section should be created for discussing relevant theories, with such theories directly linked to the hypotheses that have been formulated.

Response: Thank you for this valuable comment. We revised the theoretical background section to more clearly present the relevant theories and to link them directly to the study hypotheses.

Reviewer #6: The manuscript addresses a timely and policy-relevant question concerning how different sources of health information shape beliefs, risk perceptions, and compliance with protective behaviors during the COVID-19 pandemic in the United States. By integrating information source use, conspiracy beliefs, perceived risk, and political ideology into a single structural equation model, the study offers an ambitious attempt to connect psychological and political mechanisms of health behavior. Its strengths include the explicit theoretical grounding in Protection Motivation Theory, the use of latent-variable SEM, and the examination of both mask-wearing and vaccination intentions. At the same time, several aspects of the conceptual framing, measurement strategy, and interpretation of the modeled relationships would benefit from clearer justification and more cautious inference to strengthen the manuscript’s contribution.

1. The manuscript conceptualizes “formal” and “informal” information sources as two distinct and opposing categories (“formal information includes government websites, national television, and reports from government leaders,” while “informal information incorporates sources like social media, friends, and family members”). While analytically convenient, this dichotomy risks oversimplifying contemporary information environments, where official actors often communicate through social media and informal sources may relay institutional messages. The authors should more explicitly acknowledge this conceptual overlap and clarify whether the constructs represent source credibility, institutional trust, or communication channels.

Response: Thank you for this insightful comment. In the revised manuscript, we clarified that these constructs are intended to reflect broad source categories as operationalized in the present study rather than mutually exclusive information ecosystems. We also added text to acknowledge the conceptual overlap between communication channels, source credibility, and institutional trust, and noted this issue as a limitation when interpreting the findings.

2. The theoretical framework draws primarily on Protection Motivation Theory, emphasizing perceived severity and vulnerability as mediators of protective behavior (“threat appraisal involves the assessment of the severity of the threat and one's vulnerability to it”). However, the integration of political ideology and conspiracy beliefs into this framework remains largely additive rather than theoretically synthesized. The manuscript would benefit from a clearer articulation of how ideological orientation conceptually fits within PMT, rather than functioning as an adjacent predictor.

Response: Thank you for this helpful comment. We strengthened the theoretical framing to clarify that political ideology and conspiracy beliefs are not treated as merely adjacent predictors, but as broader interpretive factors that shape appraisals and beliefs about the effectiveness of recommended protective actions.

3. Relatedly, the manuscript’s operationalization of perceived vulnerability and susceptibility could be strengthened by engaging with work that conceptualizes perceived threat as the combined appraisal of susceptibility and severity rather than as parallel indicators. Research grounded in threat appraisal frameworks demonstrates that treating perceived threat as a joint construct, often operationalized through the interaction or composite of perceived susceptibility and perceived severity, more closely reflects how individuals evaluate health risks and translate them into behavioral responses (https://www.frontiersin.org/journals/psychology/articles/10.3389/fpsyg.2024.1332697/full). Citing this work would provide a theoretically grounded rationale for conducting a sensitivity analysis using alternative threat operationalizations, allowing the authors to assess whether their findings regarding compliance intentions are robust to different, well-established measurement approaches.

Response: Thank you for this insightful comment. We revised the manuscript to better situate perceived susceptibility and perceived severity within the broader threat-appraisal literature. Specifically, we now acknowledge that these constructs are often treated

Attachment Submitted filename: PLOS ONE Reviewer Comments.docx

# Peer review: Decision Letter 1

**DOI:** 10.1371/journal.pone.0352421.r003

**Authors:** Chao Gu

2 Jun 2026

PONE-D-24-51218R1

Why people follow or reject health guidelines: A structural equation model

PLOS One

Dear Dr. Yu,

Thank you for submitting your manuscript to PLOS ONE. After careful consideration, we feel that it has merit but does not fully meet PLOS ONE’s publication criteria as it currently stands. Therefore, we invite you to submit a revised version of the manuscript that addresses the points raised during the review process.

Please submit your revised manuscript by Jul 17 2026 11:59PM. If you will need more time than this to complete your revisions, please reply to this message or contact the journal office at plosone@plos.org. When you're ready to submit your revision, log on to https://www.editorialmanager.com/pone/ and select the 'Submissions Needing Revision' folder to locate your manuscript file.

Please include the following items when submitting your revised manuscript:

- A letter that responds to each point raised by the academic editor and reviewer(s). You should upload this letter as a separate file labeled 'Response to Reviewers'.
- A marked-up copy of your manuscript that highlights changes made to the original version. You should upload this as a separate file labeled 'Revised Manuscript with Track Changes'.
- An unmarked version of your revised paper without tracked changes. You should upload this as a separate file labeled 'Manuscript'.

If you would like to make changes to your financial disclosure, please include your updated statement in your cover letter. Guidelines for resubmitting your figure files are available below the reviewer comments at the end of this letter.

If applicable, we recommend that you deposit your laboratory protocols in protocols.io to enhance the reproducibility of your results. Protocols.io assigns your protocol its own identifier (DOI) so that it can be cited independently in the future. For instructions see: https://journals.plos.org/plosone/s/submission-guidelines#loc-laboratory-protocols. Additionally, PLOS ONE offers an option for publishing peer-reviewed Lab Protocol articles, which describe protocols hosted on protocols.io. Read more information on sharing protocols at https://plos.org/protocols?utm_medium=editorial-email&utm_source=authorletters&utm_campaign=protocols.

As the corresponding author, your ORCID iD is verified in the submission system and will appear in the published article. PLOS supports the use of ORCID, and we encourage all coauthors to register for an ORCID iD and use it as well. Please encourage your coauthors to verify their ORCID iD within the submission system before final acceptance, as unverified ORCID iDs will not appear in the published article. *Only*   the individual author can complete the verification step; PLOS staff *cannot*   verify ORCID iDs on behalf of authors.

We look forward to receiving your revised manuscript.

Kind regards,

Chao Gu

Academic Editor

PLOS One

Journal Requirements:

If the reviewer comments include a recommendation to cite specific previously published works, please review and evaluate these publications to determine whether they are relevant and should be cited. There is no requirement to cite these works unless the editor has indicated otherwise.

Please review your reference list to ensure that it is complete and correct. If you have cited papers that have been retracted, please include the rationale for doing so in the manuscript text, or remove these references and replace them with relevant current references. Any changes to the reference list should be mentioned in the rebuttal letter that accompanies your revised manuscript. If you need to cite a retracted article, indicate the article’s retracted status in the References list and also include a citation and full reference for the retraction notice.

[Note: HTML markup is below. Please do not edit.]

Reviewers' comments:

Reviewer's Responses to Questions

**Comments to the Author**

1. If the authors have adequately addressed your comments raised in a previous round of review and you feel that this manuscript is now acceptable for publication, you may indicate that here to bypass the “Comments to the Author” section, enter your conflict of interest statement in the “Confidential to Editor” section, and submit your "Accept" recommendation.

Reviewer #1: (No Response)

Reviewer #4: All comments have been addressed

Reviewer #6: All comments have been addressed

Reviewer #7: All comments have been addressed

**********

2. Is the manuscript technically sound, and do the data support the conclusions?

The manuscript must describe a technically sound piece of scientific research with data that supports the conclusions. Experiments must have been conducted rigorously, with appropriate controls, replication, and sample sizes. The conclusions must be drawn appropriately based on the data presented.

Reviewer #1: No

Reviewer #4: Yes

Reviewer #6: Yes

Reviewer #7: Yes

**********

3. Has the statistical analysis been performed appropriately and rigorously?

Reviewer #1: No

Reviewer #4: Yes

Reviewer #6: Yes

Reviewer #7: Yes

**********

4. Have the authors made all data underlying the findings in their manuscript fully available?

The PLOS Data policy requires authors to make all data underlying the findings described in their manuscript fully available without restriction, with rare exception (please refer to the Data Availability Statement in the manuscript PDF file). The data should be provided as part of the manuscript or its supporting information, or deposited to a public repository. For example, in addition to summary statistics, the data points behind means, medians and variance measures should be available. If there are restrictions on publicly sharing data—e.g. participant privacy or use of data from a third party—those must be specified.

Reviewer #1: No

Reviewer #4: Yes

Reviewer #6: Yes

Reviewer #7: Yes

**********

5. Is the manuscript presented in an intelligible fashion and written in standard English?

PLOS ONE does not copyedit accepted manuscripts, so the language in submitted articles must be clear, correct, and unambiguous. Any typographical or grammatical errors should be corrected at revision, so please note any specific errors here.

Reviewer #1: Yes

Reviewer #4: Yes

Reviewer #6: Yes

Reviewer #7: Yes

**********

6. Review Comments to the Author

Please use the space provided to explain your answers to the questions above. You may also include additional comments for the author, including concerns about dual publication, research ethics, or publication ethics. (Please upload your review as an attachment if it exceeds 20,000 characters)

Reviewer #1: The author needs to improve the formatting by ensuring proper use of capital and lowercase letters, commas, and colons. Please work on the overall formatting and writing style to enhance clarity and readability. In addition, strengthen the data analysis to make it more robust and comprehensive. The title should also be revised to make it more professional, smart, and captivating.

Reviewer #4: Authors have duly incorporated my recommendations which has significantly improved the manuscript It is now suitable for publication

Reviewer #6: The paper is improved and publishable in my opinion. I would suggest the data availability statement to be included in one of the segments of the manuscript (e.g., at the end of the manuscript).

Reviewer #7: The authors have adequately addressed the concerns raised in the previous review round. The manuscript has been substantially improved, particularly in terms of theoretical integration, clarification of limitations, and interpretation of the SEM findings. The revisions strengthened the discussion of cross-sectional design limitations, information-source categorization, and ideological pathways influencing health behaviors. The manuscript is technically sound, clearly written, and suitable for publication

**********

7. PLOS authors have the option to publish the peer review history of their article (what does this mean?). If published, this will include your full peer review and any attached files.

If you choose “no”, your identity will remain anonymous but your review may still be made public.

**Do you want your identity to be public for this peer review?**   For information about this choice, including consent withdrawal, please see our Privacy Policy.

Reviewer #1: No

Reviewer #4: **Yes:**   Antwi Joseph Barimah

Reviewer #6: No

Reviewer #7: No

**********

[NOTE: If reviewer comments were submitted as an attachment file, they will be attached to this email and accessible via the submission site. Please log into your account, locate the manuscript record, and check for the action link "View Attachments". If this link does not appear, there are no attachment files.]

To ensure your figures meet our technical requirements, please review our figure guidelines: https://journals.plos.org/plosone/s/figures

You may also use PLOS’s free figure tool, NAAS, to help you prepare publication quality figures: https://journals.plos.org/plosone/s/figures#loc-tools-for-figure-preparation.

NAAS will assess whether your figures meet our technical requirements by comparing each figure against our figure specifications.

# Author comment: Author response to Decision Letter 2

**DOI:** 10.1371/journal.pone.0352421.r004

4 Jun 2026

Please review your reference list to ensure that it is complete and correct. If you have cited papers that have been retracted, please include the rationale for doing so in the manuscript text, or remove these references and replace them with relevant current references. Any changes to the reference list should be mentioned in the rebuttal letter that accompanies your revised manuscript. If you need to cite a retracted article, indicate the article’s retracted status in the References list and also include a citation and full reference for the retraction notice.

Response: Thank you for the feedback. We carefully reviewed our reference list and ensure that it is complete and correct.

Reviewer #1: The author needs to improve the formatting by ensuring proper use of capital and lowercase letters, commas, and colons. Please work on the overall formatting and writing style to enhance clarity and readability. In addition, strengthen the data analysis to make it more robust and comprehensive. The title should also be revised to make it more professional, smart, and captivating.

Response: Thank you for this helpful suggestion. We carefully reviewed the manuscript to improve formatting. We also revised the writing throughout the manuscript to enhance clarity and readability. In addition, we revised the title to make it more professional, smart, and captivating. We agree that the data analysis should be presented in a clear and comprehensive manner. However, we believe that the current analytic approach is appropriate and sufficiently comprehensive for the aims of the study. The manuscript uses structural equation modeling to evaluate the hypothesized relationships, reports model comparison using established fit indices, presents standardized path coefficients, and includes explained variance for the endogenous variables. Given that the purpose of the study was to test the proposed structural pathways rather than to conduct additional sensitivity analyses, we retained the current analytic framework to maintain a focused and theoretically aligned presentation of the findings.

Reviewer #4: Authors have duly incorporated my recommendations which has significantly improved the manuscript It is now suitable for publication

Reviewer #7: The authors have adequately addressed the concerns raised in the previous review round. The manuscript has been substantially improved, particularly in terms of theoretical integration, clarification of limitations, and interpretation of the SEM findings. The revisions strengthened the discussion of cross-sectional design limitations, information-source categorization, and ideological pathways influencing health behaviors. The manuscript is technically sound, clearly written, and suitable for publication

Response: Thank you for the feedback. We sincerely appreciate the reviewers’ careful evaluation of the revised manuscript and are pleased that the revisions have addressed the concerns raised in the previous review round.

Reviewer #6: The paper is improved and publishable in my opinion. I would suggest the data availability statement to be included in one of the segments of the manuscript (e.g., at the end of the manuscript).

Response: Thank you for this helpful suggestion. We added the data availability statement in Results section.

Attachment Submitted filename: Response to reviewer comments v2.docx

# Editor report: Decision Letter 2

**DOI:** 10.1371/journal.pone.0352421.r005

**Authors:** Chao Gu

11 Jun 2026

Why do people comply with health guidelines in a competing information environment?

PONE-D-24-51218R2

Dear Dr. Yu,

We’re pleased to inform you that your manuscript has been judged scientifically suitable for publication and will be formally accepted for publication once it meets all outstanding technical requirements.

Within one week, you’ll receive an e-mail detailing the required amendments. When these have been addressed, you’ll receive a formal acceptance letter and your manuscript will be scheduled for publication.

An invoice will be generated when your article is formally accepted. Please note, if your institution has a publishing partnership with PLOS and your article meets the relevant criteria, all or part of your publication costs will be covered. Please make sure your user information is up-to-date by logging into Editorial Manager at Editorial Manager® and clicking the ‘Update My Information' link at the top of the page. For questions related to billing, please contact billing support.

If your institution or institutions have a press office, please notify them about your upcoming paper to help maximize its impact. If they’ll be preparing press materials, please inform our press team as soon as possible -- no later than 48 hours after receiving the formal acceptance. Your manuscript will remain under strict press embargo until 2 pm Eastern Time on the date of publication. For more information, please contact onepress@plos.org.

Kind regards,

Chao Gu

Academic Editor

PLOS One

# Editor report: Acceptance letter

**DOI:** 10.1371/journal.pone.0352421.r006

**Authors:** Chao Gu

PONE-D-24-51218R2

PLOS One

Dear Dr. Yu,

I'm pleased to inform you that your manuscript has been deemed suitable for publication in PLOS One. Congratulations! Your manuscript is now being handed over to our production team.

At this stage, our production department will prepare your paper for publication. This includes ensuring the following:

* All references, tables, and figures are properly cited

* All relevant supporting information is included in the manuscript submission,

* There are no issues that prevent the paper from being properly typeset

You will receive further instructions from the production team, including instructions on how to review your proof when it is ready. Please keep in mind that we are working through a large volume of accepted articles, so please give us a few days to review your paper and let you know the next and final steps.

Lastly, if your institution or institutions have a press office, please let them know about your upcoming paper now to help maximize its impact. If they'll be preparing press materials, please inform our press team within the next 48 hours. Your manuscript will remain under strict press embargo until 2 pm Eastern Time on the date of publication. For more information, please contact onepress@plos.org.

You will receive an invoice from PLOS for your publication fee after your manuscript has reached the completed accept phase. If you receive an email requesting payment before acceptance or for any other service, this may be a phishing scheme. Learn how to identify phishing emails and protect your accounts at https://explore.plos.org/phishing.

If we can help with anything else, please email us at customercare@plos.org.

Thank you for submitting your work to PLOS ONE and supporting open access.

Kind regards,

PLOS ONE Editorial Office Staff

on behalf of

Dr. Chao Gu

Academic Editor

PLOS One
